# A Cross-Lagged Panel Analysis of the Relationship Between Neighborhood Sense of Community and School Sense of Community

**DOI:** 10.5964/ejop.v15i4.1682

**Published:** 2019-12-12

**Authors:** Gabriele Prati, Elvira Cicognani

**Affiliations:** a Department of Psychology, University of Bologna, Bologna, Italy; London School of Economics, London United Kingdom

**Keywords:** sense of community, school, students, cross-lagged panel study

## Abstract

Neighborhood sense of community and school sense of community have been associated with important outcomes for adolescents. However, the complex interplay between neighborhood sense of community and school sense of community among adolescents is not clear. Moreover, the studies showing an association between neighborhood sense of community and school sense of community have been cross-sectional. The present study investigated the directionality of the relationship between neighborhood sense of community and school sense of community using a longitudinal cross-lagged design. Using structural equation modeling, a cross-lagged panel analysis revealed that school sense of community at Time 1 significantly predicts neighborhood sense of community at Time 2 even after controlling for neighborhood sense of community at Time 1. However, neighborhood sense of community at Time 1 did not predict school sense of community at Time 2. Results of this study support the theory that school sense of community can provide students with a bridge between school and community.

McMillan and Chavis (1986, p. 9) defined sense of community “a feeling that members have of belonging, a feeling that members matter to one another and to the group, and a shared faith that members’ needs will be met through their commitment to be together.” In the last 25 years, researchers have recognized the importance of this indicator of the perceived quality of the relationships with the community, for adolescents’ developmental outcomes and well-being (e.g., Albanesi, Cicognani, & Zani, 2007; Capone, Donizzetti, & Petrillo, 2018; Cicognani et al., 2008; Evans, 2007; Petrillo, Capone, & Donizzetti, 2016; Prati, Cicognani, & Albanesi, 2018; Pretty, Andrewes, & Collett, 1994; Pretty, Conroy, Dugay, Fowler, & Williams, 1996; Vieno, Lenzi, Santinello, & Scacchi, 2013). Sense of community in adolescence can be enhanced by shared emotional connection and positive experiences with peers and significant adults, which provide opportunities for satisfying personal needs and experiencing influence over the community (Cicognani, Zani, & Albanesi, 2012). The construct of sense of community among adolescents has been investigated mainly with reference to transactions with school and neighborhood, two of the community settings within which adolescents’ support networks are embedded and in which they share experiences and develop emotional connections with others (Cicognani, Klimstra, & Goossens, 2014; Pretty et al., 1994, 1996).

Researchers have acknowledged the role of interpersonal and societal contexts including involvement in community in adolescent development (Smetana, Campione-Barr, & Metzger, 2006). Individuals are connected to multiple communities through their multiple identities, roles, and relationships (Brodsky & Marx, 2001; Mannarini & Fedi, 2009). For instance, adolescents can develop significant relationships in one context (e.g., establishing friendships at school) contributing to their sense of community with reference to that context, and the same relationships can have spillover effects on other contexts (e.g., through sharing of other experiences in their community with classmates who are friends). The school setting is of primary relevance for adolescents in Western countries, given the significant amount of time that they spend in this context. Although it is reasonable to assume that neighborhood sense of community and school sense of community are correlated among adolescents, previous research has shown that they have distinguishing features (Chipuer, 2001; Pretty et al., 1994, 1996). Brodsky and Marx (2001) documented, in a study involving students enrolled in a job-training and education center, the presence and operation of multiple psychological senses of community with reference to multiple, separate or nested, communities. Specifically, Brodsky and Marx demonstrated the operation of quantitatively and qualitatively different multiple psychological senses of community between a macro territorial setting and a job-training and education center (which was considered a subcommunity of the territorial setting). Following these findings, we can consider the school as an example of a nested subcommunity that coexists within a territorial community toward which students may develop sense of belonging. Another difference between school sense of community and neighborhood sense of community is that adolescents tend to consider the local community as a place “not chosen” (Cicognani et al., 2012), while the school could be chosen to some extent. Choice is likely to have an influence on individuals’ psychological sense of community (Obst & White, 2007).

## Theoretical Framework

To our knowledge no previous attempts have been made to understand the complex interplay between neighborhood sense of community and school sense of community among adolescents. In the literature, we could identify two perspectives that suggest opposite paths of influence between the two aspects of sense of community. According to social disorganization theory (Shaw & McKay, 1942; Simcha-Fagan & Schwartz, 1986), the level and extent of community social organization mediate the relationship between a neighborhood’s characteristics and developmental outcomes. Social disorganization theory has been extensively utilized to explain the influence of neighborhood characteristics on youth behaviors (e.g., Bursik & Grasmick, 1993; Elliott et al., 1996; Leventhal & Brooks-Gunn, 2000; Sampson & Groves, 1989). Utilizing the social disorganization framework, research has demonstrated that community social organization (e.g., reflected in a stronger perceived sense of community) mediates the influence of negative structural characteristics on youth behaviors (e.g., Leventhal & Brooks-Gunn, 2000; Sampson & Groves, 1989; Sampson, Morenoff, & Earls, 1999). Drawing from updated systemic social disorganization models, Cantillon, Davidson, and Schweitzer (2003) provided evidence for the hypothesis that neighborhood sense of community has important spillover effects on youths’ bonding and participation in school. Therefore, based on this perspective, neighborhood sense of community could be conceptualized as a predictor of school sense of community.

The ecological model of Bronfenbrenner (1979) is specifically useful to understand youth outcomes, as it moves beyond individual characteristics to include the influence of the family and the larger sociocultural context. There is evidence that the ecological model of Bronfenbrenner (1979) can inform a deeper understanding of the factors that contribute to youth development (e.g., Bronfenbrenner, 1986; Hong, Lee, Grogan-Kaylor, & Huang, 2011; Ungar, Ghazinour, & Richter, 2013). Based on the ecological model of Bronfenbrenner (1979), Bateman (2002) suggests that school sense of community may promote sense of community with the larger communities to which the students belong. Specifically, school sense of community is associated with connections with multiple communities outside the school and opportunities for students to participate in school activities and neighborhood events. In addition, school sense of community is related to participation in accessible and diverse after-school clubs, thereby enabling students to connect with the local community. Schools can build reciprocal partnerships within the local community by contributing to the cultural and economic life of the community and by addressing its educational and health needs, for instance, through service learning initiatives (e.g., Bringle & Hatcher, 1996; Stoecker, Tryon, & Hilgendorf, 2009). Among adolescents, sense of familiarity and experiences with the context are necessary to perceive the local community as a place for experiencing feelings of belonging (Cicognani et al., 2012). In addition, there is evidence that adolescents who participate more to local community life report higher neighborhood sense of community compared to less involved adolescents (Cicognani et al., 2012). Finally, using a grounded theory approach, Cicognani et al. (2012, p. 120) showed that, among adolescents sense of community is associated with “bonding (sharing, brotherhood, acceptance, support) in the context of specific relationships (friendship, family).” The school as a community provides a place allowing a direct (face to face) contact among members. Such interactions can continue after-school and take place in the local community. In this way, a school environment that can build students’ school sense of community has the potential to promote students’ neighborhood sense of community.

One of the main limitations is the cross-sectional nature of the available studies, such that it is not possible to determine causality or direction of the associations between neighborhood sense of community and school sense of community. The aim of the present study was to examine the directionality of the association between neighborhood sense of community and school sense of community using longitudinal data and a cross-lagged panel design. Using a cross-lagged design, neighborhood sense of community and school sense of community were assessed at each time and the relations between neighborhood sense of community and school sense of community at different time points were then investigated whilst controlling for within-construct correlation (Finkel, 2004). Compared to cross-sectional design, a cross-lagged panel design enables a more accurate assessment of causality (Burkholder & Harlow, 2003). Based on the reviewed literature, we hypothesized that:

H1. According to a social-ecological approach (Bateman, 2002; Bronfenbrenner, 1979), school sense of community at Time 1 will predict neighborhood sense of community at Time 2 even after controlling for neighborhood sense of community at Time 1.

H2. According to updated systemic social disorganization models (Cantillon et al., 2003), neighborhood sense of community at Time 1 will predict school sense of community at Time 2 even after controlling for school sense of community at Time 1.

## Method

### Participants and Procedure

The procedures followed in the current study were in accordance with the ethical standards of the Italian Association of Psychology and with the 1964 Helsinki declaration. The principal of a technical institute (high school) of the Italian Region of Emilia Romagna was contacted and asked permission to conduct the study. The technical institute is co-educational (although male students predominate). Upon permission from the school, letters of consent were distributed to the students’ parents. To collect the data, we used a website accessible only to participants. Before taking part to the study, participants read a consent form that provided information about the study, instructions, and their rights as participants. The consent form made clear that participation was anonymous and voluntary. We obtained a response rate of 53% (including incomplete surveys). After obtaining informed consent, participants were asked to complete (individually) the online questionnaire during class time. Participants were approached at the beginning of the school year (Time 1) and at the end of the school year (Time 2). Between-waves attrition was 12%. There were no significant differences between participants who did and did not drop-out in terms of gender, χ^2^(1) = 0.79, *p* > .05, age, *U* = 2538.5, *p* > .05, school sense of community at T1, *U* = 2584.0, *p* > .05, and neighborhood sense of community at T1, *U* = 2530.0, *p* > .05. We linked together data from each participant at different time points using an anonymous code which was self-generated by participants. The students who were included in both Time 1 and Time 2, who thus formed the sample of the present study, numbered 106 (97 male and 9 female students). Participants’ age ranged from 13 to 17 (*M* = 14.42, *SD* = 0.67). Twenty-three participants were living in a city, 50 in a town, 26 in a village, and 7 in rural environments.

### Measures

At Time 1 and at Time 2, we asked participants to complete an online questionnaire including the measures of school sense of community and neighborhood sense of community. To measure neighborhood sense of community, we used the brief scale of Sense of Community in adolescents (Chiessi, Cicognani, & Sonn, 2010). The scale includes 20 items. Examples of items from this scale include “In this place, there are enough initiatives for young people,” “I spend a lot of time with other adolescents that live in this place,” and “People in this place support each other.” Responses were measured using a 5-point Likert type scale (1 = *not at all true*, 5 = *completely true*). The referent community was the neighborhood where the participants lived. In the current study, Cronbach’s alpha was .90 at Time 1 and .93 at Time 2.

We used the Scale of Sense of Community in the School (SoC-S; Prati, Cicognani, & Albanesi, 2017) to measure sense of community in the school. The SoC-S comprises 10 items. Examples of items from the SoC-S include “I like to stay with other students attending this school,” “In this school, I feel I can share experiences and interests with other students,” and “In this school, there are enough initiatives for me.” Participants rated the responses on a 5-point scale (1 = *not at all true*, 5 = *completely true*). In the present study, Cronbach’s alpha was .84 at Time 1 and .88 at Time 2.

### Statistical Analysis

We conducted a cross-lagged path analysis using Mplus version 7 (Muthén & Muthén, 1998–2012). We employed missing data estimation using maximum likelihood imputation procedure as recommended by Graham (2009). We tested our hypotheses using a structural equation modeling with the WLSMV estimator (a robust weighted least squares estimator using a diagonal weight matrix). An initial test of the measurement model revealed that all the latent factors were well represented by their respective indicators (i.e., all the factor loadings for the indicators on the latent variables were significant), and the overall model had a good fit, χ^2^(1704) = 2070.44, *p* < .001; NNFI = 0.91; CFI = 0.92; RMSEA = 0.045.

## Results

Table 1 displays correlations and descriptive statistics for key study variables. Neighborhood sense of community and school sense of community at Time 2 did not correlate with age and gender. Neighborhood sense of community at Time 1 correlated with gender but not with age. School sense of community at Time 1 correlated with age but not with gender. As regards intercorrelations between neighborhood sense of community and school sense of community measured at T1 and T2, all the correlation coefficients were positive and significant.

**Table 1:** Correlations Among and Descriptive Statistics for Key Study Variables

Variable	*M*	*SD*	1	2	3	4	5	6
1. Gender	-	-	-					
2. Age	16.42	0.68	−.01	-				
3. School sense of community (Time 1)	3.70	0.62	.09	−.23*	-			
4. School sense of community (Time 2)	3.43	0.79	.10	−13	.54*	-		
5. Neighborhood sense of community (Time 1)	3.34	0.63	−.33*	−.04	.26*	.34*	-	
6. Neighborhood sense of community (Time 2)	3.27	0.77	−.15	−.12	.29*	.57*	.56*	-

*Note. N*’s range from 103 to 106 due to occasional missing data. For gender, 1 = male, 2 = female.

*p < .05.

**Figure 1:**
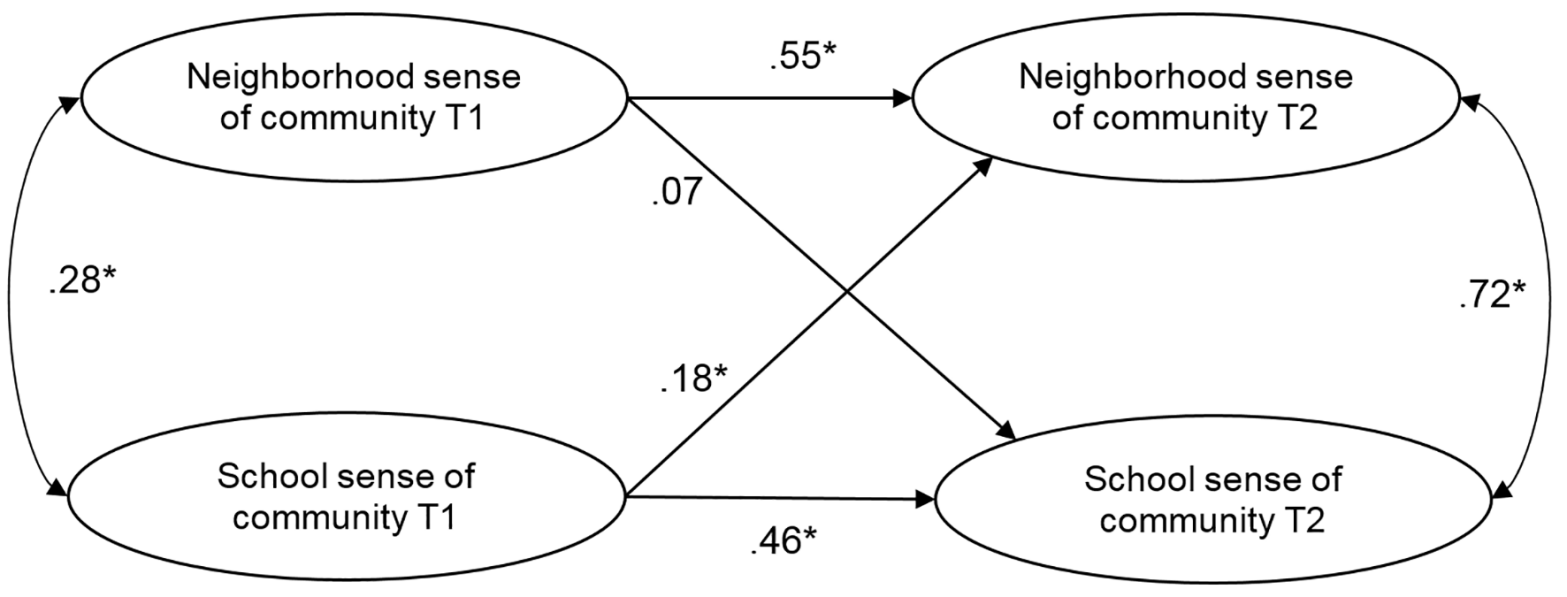
Cross-lagged relationships between neighborhood sense of community and school sense of community at Times 1 and 2 (χ^2^(1760) = 2143.67, *p* < .001; NNFI = 0.91; CFI = 0.91; RMSEA = 0.045). *Note*. Regression coefficients are standardized. *p < .05. We controlled for gender in the model.

Figure 1 displays the cross-lagged relationships between neighborhood sense of community and school sense of community at Times 1 and 2, while controlling for gender. School sense of community predicted follow up neighborhood sense of community controlling for the effects of baseline neighborhood sense of community, thereby confirming Hypothesis 1. However, T1 neighborhood sense of community did not predict school sense of community. Therefore, Hypothesis 2 was not supported by the results.

## Discussion

The purpose of this investigation was to examine the directionality of the association between neighborhood sense of community and school sense of community. With few exceptions (e.g., Prati, Albanesi, & Pietrantoni, 2016), in the literature on sense of community studies have used correlational designs to investigate this relationship (e.g., Cantillon et al., 2003; Pretty et al., 1994, 1996), despite researchers acknowledging the need for more longitudinal studies (Pretty, 2002). In the current study, we used a fully cross-lagged longitudinal design. Therefore, we were able to establish the temporal relationships between neighborhood sense of community and school sense of community.

We found that school sense of community at Time 1 predicts neighborhood sense of community at Time 2, after controlling for baseline levels of neighborhood sense of community. Thus, the data provide evidence in favor of our first hypothesis. However, in the current study, neighborhood sense of community at Time 1 did not predict school sense of community at Time 2 when controlling for school sense of community at Time 1. Therefore, the findings did not support our second hypothesis.

The results of the present study provide support for an embedded ecological model of development (Bateman, 2002). A strong sense of community in the school which is part of the microsystem is able to foster such experience in a larger social system (exosystem), such as the neighborhood community context. In the literature, several types of communities have been identified (McMillan & Chavis, 1986). For instance, there is a territorial and geographical notion of community and a relational notion of community which relates to the quality of human relationships, without reference to a place. In the present study, we demonstrated that the experience of sense of community can influence individual's sense of community with regard to another community in which the person is embedded. During adolescence, the direction of such influence seems to be from the immediate physical and social environment (microsystem) to the neighborhood community contexts (exosystem). According to the ecological framework of human development of Bronfenbrenner (1979), children may not interact directly with the exosystem; however, as they become adolescents, their interaction with the exosystem becomes more direct. Research on identity formation in adolescence revealed that the more adolescents explored different alternatives and made firm commitments in different life domains (i.e., in the achieved identity status), the more they developed neighborhood sense of community (Cicognani et al., 2014). The experiences of adolescents in their neighborhood enable the exploration of new values, roles, relationships, and interests and build civic commitment (Evans, 2007; Flanagan, Bowes, Jonsson, Csapo, & Sheblanova, 1998).

In their conceptual model, Long and Perkins (2007) defined the community social (i.e., neighboring, citizen participation, collective efficacy, informal social control, and communitarianism) and place (i.e., place attachment, community confidence, and community satisfaction) predictors of sense of community. While participation (e.g., involvement in protest activities, civic forms of engagement, public deliberation, political campaigning or voting) is not consistently associated with sense of community among adolescents (Talò, Mannarini, & Rochira, 2014), the major theoretical implication of the current research is that school sense of community may play a role in determining neighborhood sense of community among adolescents. Therefore, the role of school sense of community should be taken into account in developing conceptual models of neighborhood sense of community among adolescents. In addition, this theoretical implication bears on a practical implication, specifically on the role of the school in promoting sense of community. Indeed, these findings have practical implications for school and community psychologists. In terms of intervention programs, offering students opportunities to participate in school life, and in learning activities that stimulate collaboration within the entire school, and making the educational environment more capable to satisfy young people’s needs (i.e., to have a voice, to be heard, to develop significant relationships, to have positive experiences, to explore different options) are strategies that teachers can effectively implement in their ordinary activity to improve the relational school environment (Bateman, 2002; Whitlock, 2006) and, ultimately, students’ neighborhood sense of community. In addition, there is evidence that comprehensive, whole-school ecological intervention programs such as the Child Development Project that promote caring and supportive relationships, a sense of common purpose, cooperation between teachers, students, staff, and parents and that stimulate students’ participation in decision making can enhance students’ school sense of community (Battistich, Schaps, & Wilson, 2004).

The results of the present study should be considered in light of its limitations. First, the sample was small and not nationally representative. In addition, female participants were under-represented. Previous studies demonstrated that, compared to their male counterparts, females adolescents tend to report lower scores on neighborhood sense of community and higher scores on school sense of community (e.g., Chiessi et al., 2010; Prati et al., 2017). Although the results were controlled for gender, we cannot rule out the possibility that the findings would not generalize to other samples of students. A second limitation consists in the research design. Longitudinal research has an advantage over laboratory experiments that lack external validity; however, for determining causality, only experiments remain the gold standard. Thus, future research should be directed also toward experimental studies involving larger and more gender diverse samples.

Keeping in mind the limitations of the present study, the findings suggest that school sense of community is a temporal antecedent of neighborhood sense of community. Future research should examine interventions that address school sense of community and how these interventions may foster neighborhood sense of community. The study of the mechanisms by which school sense of community may have an influence on neighborhood sense of community is of great interest since sense of community is important for adolescents’ developmental outcomes and well-being. Although the promotion of sense of community is not a “panacea” (Prati, Albanesi, & Cicognani, 2018), more efforts should focus on understanding the experience of adolescents’ sense of community at school and in the neighborhood.

## References

[r1] Albanesi C. Cicognani E. Zani B. (2007). Sense of community, civic engagement and social well-being in Italian adolescents. *Journal of Community & Applied Social Psychology*, 17(5), 387–406. doi: 10.1002/casp.903

[r2] Bateman, H. V. (2002). Sense of community in the school. In A. T. Fisher, C. C. Sonn, & B. J. Bishop (Eds.), *Psychological sense of community: Research, applications, and implications* (pp. 161–179). Boston, MA, USA: Springer.

[r3] Battistich V. Schaps E. Wilson N. J. J. o. P. P. (2004). Effects of an elementary school intervention on students' “connectedness” to school and social adjustment during middle school. *Journal of Primary Prevention*, 24(3), 243–262. doi: 10.1023/B:JOPP.0000018048.38517.cd

[r4] Bringle R. B. Hatcher J. A. (1996). Implementing service learning in higher education. *The Journal of Higher Education*, 67(2), 221.

[r5] Brodsky A. E. Marx C. M. (2001). Layers of identity: Multiple psychological senses of community within a community setting. *Journal of Community Psychology*, 29(2), 161–178. doi: 10.1002/1520-6629(200103)29:2<161::AID-JCOP1011>3.0.CO;2-1

[r6] Bronfenbrenner, U. (1979). *The ecology of human development: Experiments by nature and design*. Cambridge, MA, USA: Harvard University Press.

[r7] Bronfenbrenner, U. (1986). Recent advances in research on the ecology of human development. In R. K. Silbereisen, K. Eyferth, & G. Rudinger (Eds.), *Development as action in context: Problem behavior and normal youth development* (pp. 287–309). Berlin, Heidelberg: Springer.

[r8] Burkholder G. J. Harlow L. L. (2003). An illustration of a longitudinal cross-lagged design for larger structural equation models. *Structural Equation Modeling: A Multidisciplinary Journal*, 10(3), 465–486. doi: 10.1207/S15328007SEM1003_8

[r9] Bursik, R. J., Jr., & Grasmick, H. G. (1993). *Neighborhoods and crime: The dimensions of effective community control*. New York, NY, USA: Lexington Books.

[r10] Cantillon D. Davidson W. S. Schweitzer J. H. (2003). Measuring community social organization: Sense of community as a mediator in social disorganization theory. *Journal of Criminal Justice*, 31(4), 321–339. doi: 10.1016/S0047-2352(03)00026-6

[r11] Capone V. Donizzetti A. R. Petrillo G. (2018). Classroom relationships, sense of community, perceptions of justice, and collective efficacy for students’ social well-being. *Journal of Community Psychology*, 46(3), 374–382. doi: 10.1002/jcop.21943

[r12] Chiessi M. Cicognani E. Sonn C. (2010). Assessing sense of community on adolescents: Validating the brief scale of sense of community in adolescents (SOC-A). *Journal of Community Psychology*, 38(3), 276–292. doi: 10.1002/jcop.20364

[r13] Chipuer H. M. (2001). Dyadic attachments and community connectedness: Links with youths' loneliness experiences. *Journal of Community Psychology*, 29(4), 429–446. doi: 10.1002/jcop.1027

[r14] Cicognani E. Klimstra T. Goossens L. (2014). Sense of community, identity statuses, and loneliness in adolescence: A cross-national study on Italian and Belgian youth. *Journal of Community Psychology*, 42(4), 414–432. doi: 10.1002/jcop.21618

[r15] Cicognani E. Pirini C. Keyes C. Joshanloo M. Rostami R. Nosratabadi M. (2008). Social participation, sense of community and social well being: A study on American, Italian and Iranian university students. *Social Indicators Research*, 89(1), 97–112. doi: 10.1007/s11205-007-9222-3

[r16] Cicognani E. Zani B. Albanesi C. (2012). Sense of community in adolescence. *Global Journal of Community Psychology Practice*, 3(4), 118.

[r17] Elliott D. S. Wilson W. J. Huizinga D. Sampson R. J. Elliott A. Rankin B. (1996). The effects of neighborhood disadvantage on adolescent development. *Journal of Research in Crime and Delinquency*, 33(4), 389–426. doi: 10.1177/0022427896033004002

[r18] Evans S. D. (2007). Youth sense of community: Voice and power in community contexts. *Journal of Community Psychology*, 35(6), 693–709. doi: 10.1002/jcop.20173

[r19] Finkel, S. E. (2004). Cross-Lagged. In M. S. Lewis-Beck, A. Bryman, & T. Futing Liao (Eds.), *The SAGE encyclopedia of social science research methods* (pp. 229–230). Thousand Oaks, CA: Sage Publications, Inc.

[r20] Flanagan C. A. Bowes J. M. Jonsson B. Csapo B. Sheblanova E. (1998). Ties that bind: Correlates of adolescents' civic commitments in seven countries. *Journal of Social Issues*, 54(3), 457–475. doi: 10.1111/0022-4537.771998077

[r21] Graham J. W. (2009). Missing data analysis: Making it work in the real world. *Annual Review of Psychology*, 60(1), 549–576. doi: 10.1146/annurev.psych.58.110405.085530 18652544

[r22] Hong J. S. Lee N. Y. Grogan-Kaylor A. Huang H. (2011). Alcohol and tobacco use among South Korean adolescents: An ecological review of the literature. *Children and Youth Services Review*, 33(7), 1120–1126. doi: 10.1016/j.childyouth.2011.02.004

[r23] Leventhal T. Brooks-Gunn J. (2000). The neighborhoods they live in: The effects of neighborhood residence on child and adolescent outcomes. *Psychological Bulletin*, 126(2), 309–337. doi: 10.1037/0033-2909.126.2.309 10748645

[r24] Long D. A. Perkins D. D. (2007). Community social and place predictors of sense of community: A multilevel and longitudinal analysis. *Journal of Community Psychology*, 35(5), 563–581. doi: 10.1002/jcop.20165

[r25] Mannarini T. Fedi A. (2009). Multiple senses of community: The experience and meaning of community. *Journal of Community Psychology*, 37(2), 211–227. doi: 10.1002/jcop.20289

[r26] McMillan D. W. Chavis D. M. (1986). Sense of community: A definition and theory. *Journal of Community Psychology*, 14(1), 6–23. doi: 10.1002/1520-6629(198601)14:1<6::AID-JCOP2290140103>3.0.CO;2-I

[r27] Obst P. L. White K. M. (2007). Choosing to belong: The influence of choice on social identification and psychological sense of community. *Journal of Community Psychology*, 35(1), 77–90. doi: 10.1002/jcop.20135

[r28] Petrillo G. Capone V. Donizzetti A. R. (2016). Classroom sense of community scale: Validation of a self-report measure for adolescents. *Journal of Community Psychology*, 44(3), 399–409. doi: 10.1002/jcop.21769

[r29] Prati G. Albanesi C. Cicognani E. (2018). The relationship between sense of community in the school and students’ aggressive behavior: A multilevel analysis. *School Psychology Quarterly*. doi: 10.1037/spq0000260 29911879

[r30] Prati G. Albanesi C. Pietrantoni L. (2016). The reciprocal relationship between sense of community and social well-being: A cross-lagged panel analysis. *Social Indicators Research*, 127, 1321–1332. doi: 10.1007/s11205-015-1012-8

[r31] Prati G. Cicognani E. Albanesi C. (2018). The impact of sense of community in the school, social skills, and exposure to aggression and victimization on students’ well-being. *Social Indicators Research*, 140(2), 637–651. doi: 10.1007/s11205-017-1808-9

[r32] Prati G. Cicognani E. Albanesi C. (2017). Psychometric properties of a multidimensional scale of sense of community in the school (SoC-S). *Frontiers in Psychology*, 8, 1466. doi: 10.3389/fpsyg.2017.01466 28900407 PMC5581826

[r33] Pretty, G. M. H. (2002). Young people’s development of the community-minded self. In A. T. Fisher, C. C. Sonn, & B. J. Bishop (Eds.), *Psychological sense of community: Research, applications, and implications* (pp. 183–203). Boston, MA, USA: Springer.

[r34] Pretty G. M. H. Andrewes L. Collett C. (1994). Exploring adolescents' sense of community and its relationship to loneliness. *Journal of Community Psychology*, 22(4), 346–358. doi: 10.1002/1520-6629(199410)22:4<346::AID-JCOP2290220407>3.0.CO;2-J

[r35] Pretty G. M. H. Conroy C. Dugay J. Fowler K. Williams D. (1996). Sense of community and its relevance to adolescents of all ages. *Journal of Community Psychology*, 24(4), 365–379. doi: 10.1002/(SICI)1520-6629(199610)24:4<365::AID-JCOP6>3.0.CO;2-T

[r36] Sampson R. J. Groves W. B. (1989). Community structure and crime: Testing social-disorganization theory. *American Journal of Sociology*, 94(4), 774–802.

[r37] Sampson R. J. Morenoff J. D. Earls F. (1999). Beyond social capital: Spatial dynamics of collective efficacy for children. *American Sociological Review*, 64(5), 633–660. doi: 10.2307/2657367

[r38] Shaw, C. R., & McKay, H. D. (1942). *Juvenile delinquency and urban areas*. Chicago, IL, USA: University of Chicago Press.

[r39] Simcha-Fagan O. M. Schwartz J. E. (1986). Neighborhood and delinquency: An assessment of contextual effects. *Criminology*, 24(4), 667–699. doi: 10.1111/j.1745-9125.1986.tb01507.x

[r40] Smetana J. G. Campione-Barr N. Metzger A. (2006). Adolescent development in interpersonal and societal contexts. *Annual Review of Psychology*, 57(1), 255–284. doi: 10.1146/annurev.psych.57.102904.190124 16318596

[r41] Stoecker, R., Tryon, E. A., & Hilgendorf, A. (2009). *The unheard voices: Community organizations and service learning*. Philadelphia, PA, USA: Temple University Press.

[r42] Talò C. Mannarini T. Rochira A. (2014). Sense of community and community participation: A meta-analytic review. *Social Indicators Research*, 117(1), 1–28. doi: 10.1007/s11205-013-0347-2

[r43] Ungar M. Ghazinour M. Richter J. (2013). What is resilience within the social ecology of human development? *Journal of Child Psychology and Psychiatry and Allied Disciplines*, 54(4), 348–366. doi: 10.1111/jcpp.12025 23215898

[r44] Vieno A. Lenzi M. Santinello M. Scacchi L. (2013). Sense of community, unfairness, and psychosomatic symptoms: A multilevel analysis of Italian schools. *Journal of Adolescent Health*, 53(1), 142–145. doi: 10.1016/j.jadohealth.2013.02.019 23623944

[r45] Whitlock J. L. (2006). Youth perceptions of life at school: Contextual correlates of school connectedness in adolescence. *Applied Developmental Science*, 10(1), 13–29. doi: 10.1207/s1532480xads1001_2

